# Soil Arthropod Diversity and Biological Soil Quality: A Web-Based Framework for Standardized EMI Attribution in QBS-ar and QBS-c

**DOI:** 10.3390/insects17070727

**Published:** 2026-07-14

**Authors:** Sara Remelli, Cristina Menta

**Affiliations:** Department of Chemistry, Life Sciences and Environmental Sustainability, University of Parma, Viale delle Scienze 11/A, 43124 Parma, Italy; sara.remelli@unipr.it

**Keywords:** soil quality, Collembola, soil arthropods, eco-morphological index, decision-support tool, standardization, bioindication, web key

## Abstract

Soil arthropods play a crucial role in soil functioning, contributing to organic matter decomposition, nutrient cycling, soil structure formation, and microbial regulation. The Soil Biological Quality index based on arthropods (QBS-ar) is widely used to assess soil quality through the eco-morphological adaptation of soil microarthropods. However, Eco-Morphological Index (EMI) attribution often relies on qualitative criteria and operator experience, highlighting the need for more standardized and transparent scoring procedures. Similar limitations affect Collembola-based approaches, where trait interpretation and score assignment may vary among users. Here, we present two web-based tools designed to support a more standardized and transparent attribution of EMI scores in QBS-ar and QBS-c. The first is an interactive identification key for major soil arthropod groups linked to explicit EMI scoring rules and automatic QBS-ar calculation. The second is a trait-based framework for Collembola (QBS-c) that translates the original rationale of Parisi’s approach into a quantitative procedure based on seven morphological traits. The QBS-c framework can also support EMI assignment for Collembola within QBS-ar when direct attribution is uncertain. The tools transform expert-based qualitative criteria into structured decision pathways, standardized trait classes, and reproducible outputs, with built-in consistency checks to improve traceability. Together, they provide a standardized and openly accessible framework for EMI assignment in soil arthropod bioindication, supporting methodological transparency, training, and future inter-operator validation of QBS-based methods.

## Simple Summary

Healthy soils are essential for food production, biodiversity, and many ecosystem services that support human well-being. Small animals living in the soil, such as mites, springtails, and other arthropods, play a key role in breaking down organic matter, recycling nutrients, and maintaining soil structure. Scientists often use these organisms to evaluate soil quality, but current assessment methods can be difficult to apply consistently because they rely heavily on expert judgement when assigning scores to different groups of organisms. This can lead to differences in results among users and studies. In this work, we developed two freely accessible online tools designed to make soil quality assessment more transparent, standardized, and easier to use. The first tool helps users identify the main groups of soil arthropods and automatically calculates a soil quality score. The second tool focuses on springtails and uses visible body characteristics to guide score assignment through a structured and reproducible process. Both tools include decision-support features that help reduce ambiguity and improve consistency. Although further testing is needed, these resources provide a practical framework for training, data harmonization, and more reliable soil biodiversity assessments, supporting future research and sustainable soil management.

## 1. Introduction

Soil microarthropods are widely recognized as effective bioindicators of soil quality due to their sensitivity to environmental conditions and their involvement in key ecological processes such as organic matter decomposition, nutrient cycling, and soil structure formation. Among the available approaches, the index based on arthropods (QBS-ar) has been extensively applied as a rapid and cost-effective method for assessing soil ecological status [1,2,3,4,5]. The QBS-ar index, first proposed by Vittorio Parisi [1], is based on the eco-morphological adaptation of soil microarthropods, assuming that higher soil quality is associated with a greater presence of taxa exhibiting traits adapted to life in the soil environment. By focusing on biological forms and levels of adaptation rather than species identity, the method enables soil quality assessment without requiring detailed taxonomic expertise, facilitating its application across a wide range of ecological and monitoring contexts. This functional, and mainly morphology-based approach (with taxonomic identification at order or class level) has supported the widespread adoption of QBS-ar in studies addressing land-use effects, environmental disturbance, and soil management practices [3]. Despite its wide application, several practical limitations affect the consistency of QBS-ar implementation. For many arthropod groups, EMI attribution is relatively straightforward once organisms are assigned to the appropriate operational category. However, difficulties arise when identification tools at low taxonomic resolution are lacking, making it challenging for non-specialist users to correctly assign specimens within the QBS framework. Although general arthropod identification keys and digital taxonomic resources are available for educational and broad biodiversity purposes (e.g., [6,7,8,9,10]), they are rarely designed specifically for soil microarthropods and do not provide a direct operational link between specimen recognition, EMI attribution, and QBS-ar calculation. In addition, even for experienced operators, multiple taxonomic keys may be required to reach the resolution needed for QBS-ar scoring, increasing procedural complexity (e.g., [11] used three different entomological keys to identify soil arthropods for QBS-ar application). Additional sources of inconsistency concern taxa for which EMI attribution is not directly inferred from group recognition alone. This is the case, for example, of adult Coleoptera, whose scoring depends on the evaluation of multiple edaphic traits, and of Collembola, where different eco-morphological conditions may correspond to different degrees of adaptation to soil life. In such cases, incomplete operational guidance may lead to inconsistent EMI attribution among users. In this context, the QBS-c index was proposed as a complementary Collembola-focused approach based on biological forms and eco-morphological adaptation rather than formal taxonomic resolution [2,12]. This is particularly relevant because Collembola include epigeic, litter-dwelling, and euedaphic forms characterized by different combinations of body size, pigmentation, eye development, appendage length, furca reduction, and cuticular structures. However, the relationship between these traits and soil adaptation is not always linear: different trait combinations may correspond to similar degrees of edaphic specialization, while intermediate or uncommon morphologies may complicate direct EMI attribution. Recent syntheses have also highlighted substantial heterogeneity in trait selection, scoring schemes, and QBS-c calculation across studies, emphasizing the need for clearer operational rules for Collembola EMI attribution [13]. Functional traits and associated functional groups are increasingly employed to investigate how Collembola communities respond to environmental changes and contribute to ecosystem functioning [14] and, for QBS-c, attempts to address the previously mentioned issues have included simplifying trait contributions and excluding selected characters in trait-based approaches [15]. However, such simplifications may reduce the resolution of the system, potentially flattening differences among eco-morphological forms compared to the original framework proposed by Parisi [1]. Thus, although QBS-c provides a biologically coherent framework, its practical application still requires a more explicit translation of morphological traits into standardized scoring procedures. Beyond identification and scoring, discrepancies may also emerge during index calculation, for example, when EMI values are summed incorrectly or when the rule of retaining the highest EMI value per taxon is not consistently applied. Consequently, part of the variability observed among QBS-ar applications may reflect methodological differences rather than ecological variation alone. These limitations highlight the need for clearer operational tools that support consistent identification, explicit and standardized EMI attribution, and transparent index calculation procedures. This need is consistent with broader developments in biodiversity monitoring and with the recent European policy framework established by Directive (EU) 2025/2360 on soil monitoring and resilience, which further emphasizes the importance of harmonized protocols, explicit decision rules, and traceable workflows for the operational application of biological soil quality indicators [16,17,18]. Observer-dependent variability is a well-recognized source of bias in biodiversity monitoring, particularly when identification and classification rely on individual expertise and judgement [19]. In ecological assessment, decision-support systems can help translate expert knowledge, indicators, and thresholds into more transparent and operational workflows [20]. Recent efforts have addressed this need by providing standardized workflows, predefined biological forms, and data recording templates for QBS-ar application [21,22]. However, these approaches primarily focus on standardizing data recording and classification outputs, while EMI attribution itself often remains dependent on implicit trait interpretation and operator judgement. The reliance on expert-based interpretation, combined with the absence of explicit decision rules, makes it difficult to assess whether EMI attribution is applied consistently across studies, as the underlying scoring logic is rarely documented. The recent adoption of Directive (EU) 2025/2360 [18] on soil monitoring and resilience further strengthens the relevance of QBS-based approaches. By establishing a common framework for soil health monitoring across the European Union, the Directive explicitly incorporates biological soil quality indicators, including approaches based on soil biodiversity and biological activity, among the elements to be considered in soil assessment. QBS-ar and QBS-c are directly relevant to this context because they provide rapid, morphology-based information on soil arthropod communities and their adaptation to soil life. Their inclusion in, or alignment with, European soil monitoring frameworks makes the standardization of EMI attribution a critical operational issue. Indeed, the reliability and comparability of QBS-based indicators depend on the consistent translation of morphological observations into eco-morphological scores. The framework proposed here addresses this requirement by providing explicit decision rules, traceable scoring pathways, and standardized outputs, thereby supporting the practical implementation of QBS-based indicators within European soil monitoring programmes.

Here, we present two web-based tools designed to formalize EMI attribution in QBS-ar and QBS-c. The first is an interactive QBS-ar identification key that links diagnostic morphological decisions to predefined EMI rules and automatic index calculation. The second is a trait-based QBS-c framework that converts Collembola morphology into standardized score combinations and QBS-ar-compatible EMI classes. The novelty of the framework lies in the explicit connection between morphological observation, EMI attribution, decision-pathway documentation, and exportable outputs. Rather than proposing a new soil quality index, the tools operationalize existing QBS principles in a structured and traceable workflow intended to support training, comparison among users, and future inter-operator validation.

## 2. Materials and Methods

### 2.1. Framework Structure and Workflow

The framework consists of two complementary client-side web tools: an interactive QBS-ar identification and scoring module, and a trait-based QBS-c module for Collembola. The two modules can be used independently or in an integrated workflow. In the integrated workflow, the QBS-c module provides trait-based support for EMI attribution in Collembola when qualitative classification within the QBS-ar key is uncertain.

The methodological structure of the framework follows four sequential components: (i) operational identification of QBS-ar taxa through a hierarchical decision key; (ii) attribution of fixed or variable EMI values according to predefined taxon-specific rules; (iii) trait-based scoring of Collembola using the QBS-c module; and (iv) standardized recording, calculation, and export of QBS-ar and QBS-c outputs. This structure was used to organize the development and description of the two tools.

### 2.2. QBS-ar Interactive Identification Key

The QBS-ar tool was implemented as a hierarchical identification key based on progressive node navigation. The workflow guides users from general morphological criteria (e.g., body organization, segmentation, appendage structure) to terminal operational taxa through a sequence of binary or multiple-choice decisions. The key includes 27 decision nodes and covers the major arthropod groups typically encountered in soil samples. The identification workflow is complemented by a web-based Soil Arthropod Atlas, which provides standardized visual references, diagnostic characters, ecological information, and EMI-associated biological forms for the major arthropod groups included in the QBS-ar framework. The Atlas can be consulted independently or in parallel with the identification key, serving as a training and decision-support resource for both novice and experienced users.

Illustrative images are provided for morphological characters and terminal taxa whenever available, to support interpretation of diagnostic features.

Each terminal taxon is associated either with a fixed EMI value or with a restricted set of alternative values reflecting intra-group eco-morphological variability (Table S1). The decision logic therefore operates in two sequential steps. First, diagnostic morphological characters guide the user to an operational QBS-ar group or eco-morphological category. Second, the tool assigns either a fixed EMI value or a constrained set of EMI options according to the scoring rules allowed for that group. For variable-score taxa, EMI attribution is restricted to predefined morphological criteria, preventing the use of unconstrained or non-standard values.

The complete list of fixed-score groups, variable-score groups, allowed EMI values, and special attribution rules is provided in Table S1. The key was designed to cover the operational QBS-ar groups and eco-morphological categories implemented in the standard index, including both fixed-score taxa and groups requiring variable EMI attribution. The full decision-tree structure, including terminal nodes and corresponding outputs, is reported in Table S2.

For Collembola, the key includes ordered ecological categories ranging from epigeic to euedaphic forms, with a distinct class for clearly euedaphic organisms assigned the maximum EMI value. Adult Coleoptera are treated through a cumulative rule in which multiple edaphic traits contribute additively to the final EMI attribution.

The identification key is linked to an integrated calculator that stores taxa, selected EMI values, and abundance. Abundance is recorded as complementary quantitative information and does not contribute to the calculation of the standard QBS-ar score, which is based on the highest EMI value retained for each operational taxon. In the current implementation, abundance documents the number of individuals or records associated with each taxon, supports total-abundance summaries, and enables subsequent complementary analyses, including abundance-based QBS extensions such as QBS-ab and other biodiversity indices. For taxa identified through the guided key, the interface also records the decision pathway followed by the user. This pathway reports the sequence of selected diagnostic nodes, including the node number, the diagnostic question, and the selected option. It can be inspected during the identification process and is exported together with the corresponding taxon, EMI value, and abundance record. Direct taxon entry remains available for expert users; in this case, no decision pathway is generated because the identification is performed independently of the interactive key.

The QBS-ar index is calculated by retaining the highest EMI value per taxon and summing across taxa, following the original method. The QBS-ar table is updated dynamically after each taxon entry. Abundance is initialized at one for each new record and can be subsequently modified by the user. Total abundance and the final QBS-ar value are recalculated automatically, with the QBS-ar score obtained by retaining the highest EMI value per operational taxon and summing across taxa according to the standard QBS-ar rule. Outputs are generated in a standardized tabular format and can be exported for further analysis.

### 2.3. Trait-Based QBS-c Framework for Collembola

The QBS-c module assigns EMI values to Collembola using seven morphological traits: body size, pigmentation, visual apparatus, antennae length, leg length, furca development, and cuticular structures (Table 1).

Each trait is divided into ordered classes representing increasing levels of adaptation to soil life. These traits were selected because they represent the core eco-morphological characters traditionally used in the QBS-c approach to describe adaptation to soil life, including reduction in pigmentation and visual structures, shortening of appendages, modification or loss of the furca, and changes in cuticular structures. The present framework does not introduce a new trait system; rather, it formalizes the characters of the original QBS-c rationale into ordered classes and explicit numerical scores, so that trait interpretation and EMI attribution can be applied through a reproducible rule-based procedure.

The total QBS-c value is calculated as the sum of the seven trait scores, transforming a qualitative eco-morphological gradient into an additive index. Each combination is encoded as an ordered score sequence, ensuring that the full trait profile is preserved and traceable. Abundance is recorded in the QBS-c tool as complementary quantitative information and is not used as a weighting factor in the calculation of the trait-based QBS-c score. Repeated entries of the same trait combination are aggregated to document how many individuals share the same eco-morphological profile, while preserving each combination as a unique scoring unit. This structure allows the output to be used for community-level summaries, complementary biodiversity analyses, and future extensions of the tool. To ensure compatibility with QBS-ar, total QBS-c scores are converted into QBS-ar-compatible EMI classes following the relationship proposed in the original QBS-c framework, in which the corresponding QBS-ar value is obtained by dividing the QBS-c score by two. Because QBS-ar uses discrete EMI classes for Collembola, the resulting value is then assigned to the corresponding class implemented in the tool.

This mapping allows the trait-based framework to function as an operational bridge between continuous scoring and categorical EMI attribution.

A rule-based consistency tool was incorporated to detect biologically implausible trait combinations. The system evaluates the coherence of selected traits and flags inconsistent profiles (e.g., large body size associated with strongly reduced appendages, or combinations of well-developed epigeic traits with strong reduction in locomotory structures).

Natural Collembola assemblages may include rare, intermediate, or atypical trait combinations that do not fit neatly into predefined eco-morphological categories. For this reason, the consistency check is intended as a diagnostic support rather than as an automatic correction procedure. Flagged combinations are not excluded, and their scores are not modified; instead, the user is prompted to re-examine the selected traits and, when necessary, to rely on expert judgement or additional taxonomic information. This approach preserves the transparency of the scoring process while acknowledging that some biologically realistic combinations may remain difficult to classify unambiguously.

The QBS-c tool can be used either as a standalone tool for full Collembola scoring or as a decision-support system within QBS-ar. In the latter case, trait-based scoring provides an explicit reference for EMI assignment when qualitative classification is uncertain, ensuring greater transparency and coherence between the two approaches.

### 2.4. Algorithmic Implementation and Output Structure

Both tools were implemented as standalone client-side web applications using HTML, CSS, and JavaScript, ensuring accessibility and transparency without requiring server-side computation. The system dynamically renders decision nodes, calculates scores in real time, and stores user-defined entries in cumulative tables. Outputs are fully traceable and exportable in standardized formats.

The tool was developed as part of the present study and is currently made available in association with this manuscript at: https://www.soilarthropo-lab.unipr.it/index.php/qbs-ar-identification-key/ (accessed on 9 July 2026). It represents a structured methodological proposal based on expert knowledge and literature-derived criteria.

In addition to the guided identification workflow, the tool allows direct registration of taxa that have already been identified by the user. This functionality is intended for experienced operators and laboratories that perform taxonomic identification independently of the interactive key. Taxa can be entered directly into the cumulative table, where EMI values and QBS-ar calculations are automatically managed according to the standard QBS-ar rules. This dual workflow enables the tool to function both as an identification aid and as a standardized QBS-ar calculation environment.

## 3. Results

### 3.1. Structure and Functionality of the QBS-ar Interactive Key

The QBS-ar tool provides a fully navigable identification workflow structured as a hierarchical decision tree, where most decision nodes are supplemented with representative images or visual references to support interpretation of diagnostic morphological characters.

The navigation system retains the history of previous steps, allowing users to revise earlier decisions, and enables repeated identification cycles through a restart function. The guided workflow also includes a visible decision-pathway panel. This panel records the sequence of diagnostic decisions made by the user, reporting for each step the node number, the diagnostic question, and the selected option. The pathway remains available for inspection during the identification process and is associated with the corresponding output record when the taxon and EMI value are added to the QBS-ar table. Upon reaching a terminal node, the corresponding taxon is automatically linked to its EMI value or to a set of predefined EMI options. These values can be directly recorded in the QBS-ar table, where each entry is stored together with its taxonomic identity and user-defined abundance. The system therefore separates identification, EMI attribution, and data recording into distinct but integrated steps. The full node-based structure of the identification key, including all decision steps and outputs, is provided in Table S2, ensuring complete transparency and reproducibility of the classification workflow.

### 3.2. Implementation of EMI Attribution Rules in the QBS-ar Tool

**Figure 1:**
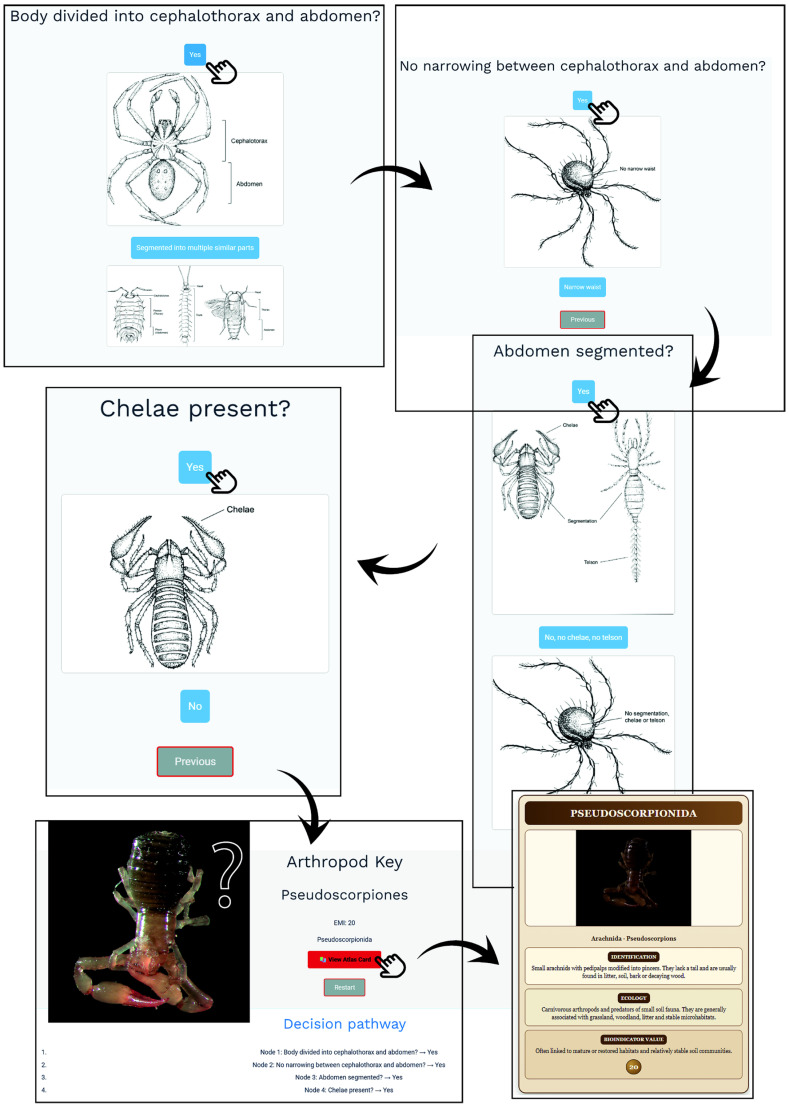
QBS-ar interactive identification key and traceable decision pathway, using as an example the identification of the organism in the photo placed at the bottom-right of the figure. Once a terminal taxon is reached, the interface provides the associated EMI value and a direct link to the corresponding Soil Arthropod Atlas card for additional diagnostic and ecological information.

The QBS-ar tool operationalizes EMI attribution through a combination of fixed values and rule-based options. Fixed EMI values are automatically assigned to taxa considered operationally homogeneous (Figure 1), whereas groups with known eco-morphological variability are handled through explicit user-selected options.

Figure 1 illustrates the guided identification workflow and the decision-pathway panel implemented in the QBS-ar module. The figure shows how diagnostic choices are recorded as an inspectable sequence linking morphological observation to terminal taxon assignment and EMI attribution. The direct connection with the Soil Arthropod Atlas provides additional visual and diagnostic support for the selected taxon.

**Figure 2:**
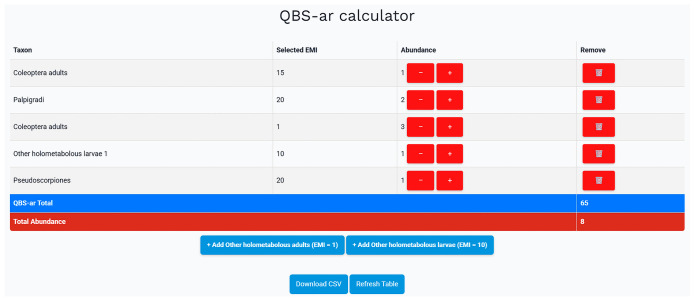
QBS-ar calculator interface and automatic output generation. For each entry, the table reports the operational taxon, the selected EMI value, and abundance, which can be adjusted manually using the plus and minus controls or removed from the dataset. The final QBS-ar value is calculated automatically, while total abundance is updated dynamically after each entry or modification. Additional buttons allow the user to record unresolved holometabolous adults or larvae with predefined EMI values and to export the complete table in CSV format.

The lower panel displays the decision-pathway panel that records the sequence of diagnostic nodes selected by the user, including the node number, diagnostic question, and selected option. Figure 2 shows the integrated QBS-ar calculator, where identified taxa are automatically recorded together with their associated EMI values.

Identified taxa are stored together with selected EMI values and abundance, while the final QBS-ar score is calculated automatically according to the standard rule of retaining the highest EMI value for each operational taxon. The exportable output links the recorded taxon, selected EMI value, abundance, and, for guided identifications, the associated decision pathway.

Overall, Figure 1 and Figure 2 illustrate the integration of guided taxon identification, EMI attribution, abundance recording, and automatic QBS-ar calculation within a single workflow. The decision-pathway panel documents the sequence of diagnostic nodes selected by the user, making the route from morphological observation to terminal taxon inspectionable before the record is added to the QBS-ar table. The corresponding output can then be exported together with the selected EMI value and abundance, supporting traceability from the decision process to the final index calculation.

The implemented rule sets distinguish between fixed-score taxa and groups requiring constrained EMI selection. For fixed-score groups, the EMI value is assigned automatically once the terminal taxon is reached. For variable-score groups, including Collembola and adult Coleoptera, the tool restricts attribution to predefined eco-morphological options or cumulative trait-based rules. This structure prevents unconstrained score assignment and makes the criteria used for EMI attribution explicit in the output workflow.

### 3.3. Structure and Outputs of the QBS-c Trait-Based Tool

**Figure 3:**
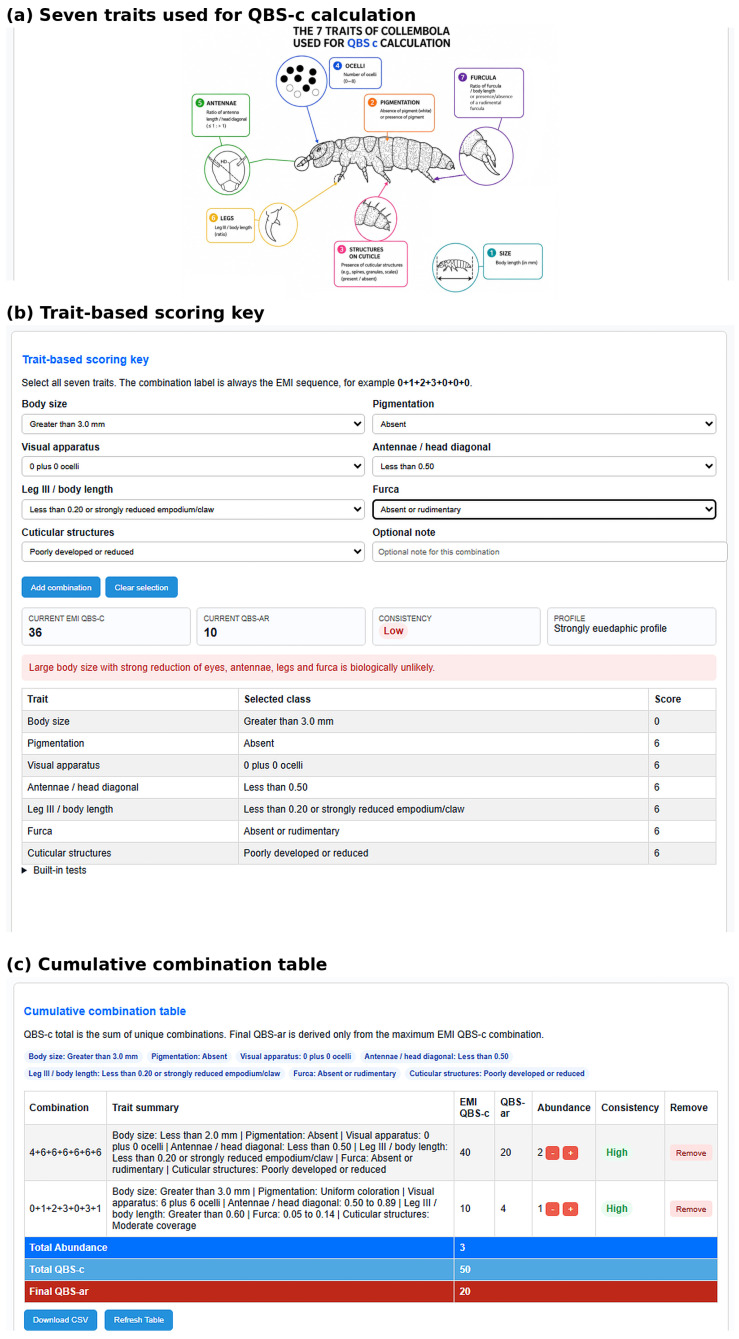
Interface of the QBS-c trait-based tool showing (**a**) the seven morphological traits considered for QBS-c calculation, (**b**) the trait-based scoring key used to calculate the QBS-c value and (**c**) the cumulative combination table used to derive the final QBS-ar score.

The QBS-c tool provides a structured trait-based interface for scoring Collembola based on seven morphological characters (Figure 3a). Users are required to complete all trait fields before the system calculates the score, ensuring that each output corresponds to a fully defined eco-morphological profile.

For each completed combination, the tool generates:

- The total QBS-c score;
- The corresponding QBS-ar-compatible EMI class;
- A trait-by-trait breakdown of the score;
- A compact identifier representing the trait combination;
- A qualitative consistency assessment;
- A simplified eco-morphological profile descriptor.

The compact identifier preserves the complete trait-score sequence and facilitates comparison among repeated or similar eco-morphological profiles. The qualitative consistency assessment functions as a quality-control layer by flagging trait combinations that deviate from the expected covariation among morphological characters. The eco-morphological profile descriptor provides a simplified ecological interpretation of the combination, distinguishing predominantly epigeic, intermediate, and strongly euedaphic profiles. All outputs are displayed in real time and can be stored in a cumulative table (Figure 3).

Repeated entries of the same trait combination are aggregated in the cumulative table, increasing the associated abundance value rather than duplicating the profile. Thus, each trait configuration remains uniquely identifiable while retaining quantitative information for complementary analyses.

### 3.4. Internal Consistency Assessment and Profile Classification

The QBS-c tool includes an internal consistency-checking system that evaluates the plausibility of trait combinations that conform to expert-defined eco-morphological patterns embedded in the tool. These patterns are based on the expected covariation among traits associated with surface-dwelling and soil-adapted forms, such as pigmentation, visual apparatus, appendage length, and furca development. When all seven traits are selected, the system automatically assesses whether the selected trait combination is consistent with expected eco-morphological patterns.

Combinations identified as inconsistent are flagged and accompanied by an explanatory message indicating the nature of the mismatch (Figure 3b). For example, a large body size combined with simultaneous strong reduction in eyes, antennae, legs, and furca is flagged as unlikely, as is a combination of fully epigeic traits with strongly reduced locomotory structures. These flags are displayed in real time and recorded in the output table, thereby providing a transparent diagnostic layer without modifying the calculated score.

In parallel, each combination is assigned to a simplified eco-morphological profile based on the relative expression of key functional traits (visual apparatus, antennae, legs, and furca). This classification distinguishes predominantly epigeic, intermediate, and strongly euedaphic profiles.

### 3.5. Output Structure and Data Export

Both tools generate structured tabular outputs that can be exported in CSV format. The QBS-ar output includes taxa, selected EMI values, and abundance, together with the final QBS-ar score. The QBS-c output includes trait combinations, scores, consistency flags, profile classification, and abundance, as well as cumulative QBS-c and derived QBS-ar values.

This standardized output format ensures full traceability of the scoring process, from initial identification or trait selection to final index calculation, and allows subsequent analysis, comparison among operators, or integration with external datasets.

### 3.6. Integration Between QBS-c and QBS-ar for Collembola

The integration of the QBS-c tool with the QBS-ar framework enables a consistent treatment of Collembola across both approaches. For each trait-based combination, the total QBS-c score is automatically converted into a QBS-ar-compatible EMI class following the relationship proposed in the original QBS-c framework, in which the corresponding QBS-ar value is obtained by dividing the QBS-c score by two. Because QBS-ar uses discrete EMI classes for Collembola, the resulting value is then rounded and assigned to the corresponding QBS-ar-compatible class implemented in the tool. Accordingly, the conversion follows these intervals: 0–3 = EMI 1, 4–7 = EMI 2, 8–10 = EMI 4, 11–14 = EMI 6, 15–19 = EMI 8, 20–36 = EMI 10, and ≥37 = EMI 20. This rule preserves the link with the original QBS-c rationale while allowing the trait-based output to be directly integrated into the discrete EMI structure of QBS-ar.

This mapping allows the trait-based output to be directly incorporated into the QBS-ar logic, where the highest EMI value is retained for the Collembola group. In practical terms, the QBS-c tool can therefore be used to support EMI attribution in cases where qualitative classification within the QBS-ar key is uncertain.

The integration ensures that both tools operate within a coherent scoring framework while maintaining their respective methodological approaches.

### 3.7. Example Application of the Integrated Workflow

**Figure 4:**
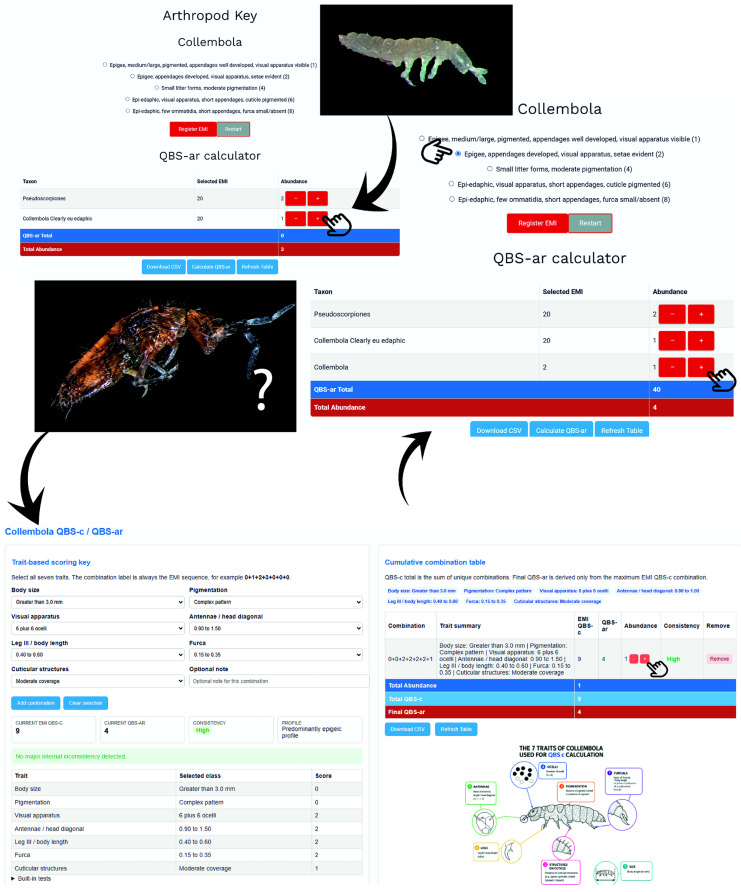
QBS-c trait-based scoring tool and integration with QBS-ar.

The operational workflow of the tool is summarized in Figure 4. Figure 4 illustrates the QBS-c trait-based tool and its integration with the QBS-ar workflow through a representative example. The interface is organized into a trait selection panel (left) and a cumulative results table (right). The process begins with the identification of arthropod taxa using the QBS-ar key, followed by EMI attribution based on predefined rules. For Collembola, the QBS-c tool can be used to generate a trait-based score and corresponding EMI class when direct classification is uncertain.

The resulting EMI values are then integrated within the QBS-ar framework, where the highest value per taxon is retained and summed to produce the final index. The tool thus combines identification, scoring, and output generation within a single traceable workflow.

The example shows how the QBS-c module can provide an explicit trait-based score and a corresponding QBS-ar-compatible EMI class for ambiguous Collembola forms. When QBS-c is used only as support for QBS-ar, selected trait combinations can be entered to guide EMI attribution. Conversely, when the objective is to calculate a complete QBS-c index, all Collembola occurrences must be entered into the QBS-c module so that cumulative QBS-c and derived QBS-ar outputs are based on the complete set of trait combinations.

## 4. Discussion

### 4.1. Practical Value of the Proposed Tools

The tools presented here are designed to support EMI attribution within QBS-based soil biological assessment. By integrating identification, scoring, and data recording into a single workflow, the tool facilitates the application of both QBS-ar and QBS-c in research and applied contexts. The use of structured decision nodes and standardized outputs improves traceability of the scoring process and may support training, inter-operator comparison, and data harmonization across studies. Moreover, the possibility to record organism counts and export data in CSV format allows users to apply QBS-ar extensions (e.g., QBS-ab; [23]) as well as additional biodiversity indices.

Importantly, the system is not intended to replace expert judgement, but to make the decision process more explicit, inspectable, and reproducible. This is particularly relevant in contexts where taxonomic expertise is limited or where rapid and standardized assessments are required.

Beyond guided identification, the QBS-ar module also functions as a standardized recording and calculation environment. Users who have already completed taxonomic identification can bypass the identification workflow and directly register taxa, allowing EMI values and QBS-ar calculations to be managed according to predefined rules. The integration of the Soil Arthropod Atlas further extends the educational value of the framework. By providing visual references, diagnostic characters, and ecological notes linked to the operational taxa used in QBS-ar, the Atlas may support user training and reduce interpretation errors during specimen recognition, particularly for non-specialist users or laboratories applying QBS-based methods in routine monitoring.

### 4.2. Standardization, Transparency, and Consistency in EMI Attribution

The main methodological contribution of the proposed framework is not only the digital implementation of QBS-ar and QBS-c procedures, but the explicit formalization of the decision logic linking morphological observation, EMI attribution, and the final index calculation. This issue has recently been emphasized by Coletta et al. [13], who documented substantial methodological variability in trait selection, scoring direction, EMI assignment, and QBS-c aggregation procedures across studies. In many current applications of QBS-ar, EMI assignment still depends on implicit knowledge, general descriptions, or broad group-level assumptions, which may increase operator dependency and reduce reproducibility.

Previous initiatives have contributed to improving the standardization of QBS-ar applications by defining biological forms, structured sampling protocols, and standardized data-recording tools [22]. These efforts represent an important step toward harmonization. However, they do not fully resolve the ambiguity associated with EMI attribution itself, which often remains dependent on expert interpretation, nor do they provide an operational identification pathway linking specimen recognition to score assignment. The approach proposed here addresses both aspects by integrating a hierarchical identification key with an explicit scoring framework, linking identification, EMI attribution, and index calculation within a traceable workflow.

By embedding scoring options within explicit decision pathways and standardized output tables, the tool makes the attribution process more transparent and operationally constrained. While interpretation remains necessary, the extent to which scoring decisions are implicit is substantially reduced.

An additional issue emerging from the literature concerns QBS-ar values that are difficult to reconcile with the structural logic of the index [24,25,26]. Because QBS-ar is calculated as the sum of EMI values bounded between 1 and 20, the final score is constrained by both the number of taxa recorded and the maximum value assignable to each operational unit. Although no universal upper threshold can be defined a priori, the index remains biologically bounded by the number of eco-morphologically distinct taxa co-occurring in a sample. Some published values appear difficult to interpret in relation to these constraints. Such cases may reflect differences in taxonomic resolution, aggregation criteria, EMI attribution practices, or departures from the standard calculation procedure rather than ecological conditions alone. Conversely, very low values in samples that contain several soil-adapted groups may indicate incomplete extraction, restricted taxonomic coverage, or deviations from standard scoring procedures. These interpretations should be considered cautiously because ecological conditions, sampling intensity, and habitat type can strongly influence the expected range of QBS-ar values. Taken together, these patterns suggest that part of the variability reported in the literature may be methodological as well as ecological, reinforcing the need for explicit and standardized scoring procedures.

### 4.3. Deviations from Standard EMI Scoring Rules

Beyond variability in the final index values, inconsistencies also emerge at the level of EMI attribution (e.g., [24,27,28,29]). In some cases, EMI values exceeding the theoretical maximum of the QBS-ar framework (i.e., >20) have been reported, resulting in QBS-ar scores that are not directly comparable with standard applications of the index. In other cases, departures from the original rule structure occur within specific taxonomic groups. For example, some groups may be assigned values that do not conform to the expected discrete scoring scheme, while others may receive values lower than those implied by their strongly edaphic morphology. Likewise, the use of broad and unconstrained score ranges for entire groups, without explicit morphological criteria, obscures how scores are actually assigned. Recent reviews of QBS-c applications have shown that even the directionality of EMI scoring is not applied consistently across studies, with some frameworks assigning higher values to surface-adapted forms rather than to euedaphic specialization [13]. These deviations appear to be relatively uncommon rather than systematic across the literature. However, their occurrence is methodologically relevant because even isolated departures from the standard EMI scale or from the original aggregation rules can reduce comparability among studies and complicate the interpretation of QBS-based assessments. These patterns do not necessarily imply errors in individual studies, but they do indicate substantial heterogeneity in the operational implementation of QBS-ar. This heterogeneity reduces comparability among applications and highlights the need for clearer constraints linking morphology, biological form, and score attribution.

In this respect, the present framework is designed to reduce such ambiguity by embedding discrete scoring rules within a guided decision process. Each EMI value is linked to explicit morphological criteria, making the logic of score attribution inspectable and more consistent across applications.

### 4.4. Specific Advantages of the QBS-c Framework for Collembola

The trait-based QBS-c tool addresses a particularly critical component of QBS-ar applications, namely EMI attribution in Collembola. This group encompasses a wide range of eco-morphological adaptations, and its classification into discrete EMI categories is often based on qualitative interpretation.

Previous studies [30] have explored the use of Collembola morphological traits as soil quality indicators, showing that trait-based or morphotype-based approaches can provide a practical alternative to full taxonomic identification. However, these applications often rely on adapted scoring schemes, study-specific trait selections, or alternative calculation procedures, which may limit direct comparability across studies. In this context, the framework proposed here differs by explicitly formalizing trait classes, score attribution, and QBS-ar compatibility within a transparent and reproducible decision-support structure.

In the broader literature (e.g., [31,32,33]), QBS-c is frequently applied in association with species- or genus-level identification. Although this may increase ecological resolution, it also introduces a degree of taxonomic dependency that is not strictly required by the conceptual basis of QBS indices. The original rationale of the QBS approach is grounded in eco-morphological adaptation rather than taxonomic identity and was conceived to support relatively rapid and accessible soil quality assessment without requiring species-level expertise.

By formalizing trait-based scoring through an explicit and constrained framework, the proposed QBS-c tool aims to realign EMI attribution with its eco-morphological foundations while reducing dependence on taxonomic resolution.

The inclusion of internal consistency checks adds a diagnostic layer by identifying trait combinations that are unlikely according to the expected covariation among eco-morphological characters. For example, a profile combining a large body size with complete loss of eyes, strongly reduced antennae and legs, and an absent or rudimentary furca is flagged as unlikely because these strongly edaphic reductions are generally expected to occur together with other forms of miniaturization or soil-adapted morphology. Similarly, combinations of fully epigeic traits, such as well-developed eyes and furca, with strongly reduced locomotory appendages are flagged for re-examination. These warnings do not modify the score but prompt the user to verify the selected traits and, if necessary, consult additional taxonomic information. While recent efforts have advocated simplified trait subsets to facilitate routine application of QBS-c [13], the present framework deliberately retains the full seven-trait architecture originally proposed by Parisi [12]. The objective is not to simplify eco-morphological interpretation by reducing trait dimensionality, but rather to standardize and formalize the original conceptual structure through explicit decision rules and reproducible scoring procedures.

The integration between QBS-c and QBS-ar represents a potentially useful feature of the system. Trait-based scoring may support EMI attribution when qualitative classification within QBS-ar is uncertain, thereby bridging categorical classification and explicit eco-morphological evaluation. This is particularly relevant for intermediate forms that are difficult to classify using traditional approaches.

### 4.5. Limitations

The main limitation of the present work is that the tool has not yet undergone formal usability testing or inter-operator validation across independent users and institutions. Its routine use within our laboratory has supported iterative refinement of the workflow, but this internal application should not be interpreted as a substitute for formal external validation. Therefore, these expected benefits should currently be regarded as methodological objectives rather than empirically validated outcomes.

In addition, although the rule-based framework reflects current knowledge of eco-morphological adaptation, it necessarily involves a degree of abstraction and simplification. Some decision rules and scoring thresholds may require further refinement as additional empirical evidence and user-feedback data become available.

### 4.6. Perspectives

Future work should move from internal methodological development to formal external validation of the tool across users, laboratories, and institutional contexts, with particular attention given to differences in taxonomic expertise and previous experience with QBS-based methods.

Comparative studies between traditional and tool-assisted scoring approaches would be particularly valuable. Beyond single-user or single-laboratory testing, a key future step should be the organization of a coordinated inter-laboratory validation study involving research groups and technical laboratories already applying QBS-based methods. Such a study should include users with different levels of taxonomic and methodological expertise and should compare traditional EMI attribution with tool-assisted workflows using shared reference samples, image sets, or standardized digital case studies. This approach would allow the reproducibility, inter-operator consistency, usability, and practical added value of the framework to be quantified under realistic application conditions. At the same time, a collaborative validation network could support broader adoption of the tool and contribute to the harmonization of QBS-ar and QBS-c procedures across the scientific and technical community.

Further developments may include automated image recognition for preliminary taxon or trait screening, integration with soil biodiversity databases, and connection with environmental monitoring platforms. These developments would extend the framework beyond a standalone decision-support tool and could support broader adoption of standardized QBS-based bioindication in research, technical monitoring, and European soil health assessment.

## 5. Conclusions

This study presents a methodological framework for formalizing EMI attribution in QBS-ar and QBS-c through explicit decision rules, standardized trait classes, and traceable outputs. Its main contribution is not only the digital implementation of QBS-based procedures, but the operational translation of expert-based qualitative criteria into inspectable scoring pathways.

By integrating a QBS-ar identification key with a trait-based QBS-c module for Collembola, the framework provides a common structure for linking morphological observations, EMI attributions, and final index calculations.

At the current stage, the tool should be regarded as a structured decision-support tool rather than a formally validated diagnostic system. Its next developmental step is formal validation across users with different levels of taxonomic expertise, in order to assess its effectiveness in improving consistency, transparency, and comparability in QBS-based applications. Future inter-laboratory validation will be necessary to assess its effect on inter-operator consistency, usability, and comparability across datasets and monitoring contexts.

Overall, the framework may contribute to the standardization and comparability of QBS-based soil bioindication by making the rules used for EMI attribution more explicit, inspectable, and accessible to both scientific and technical users.

## Figures and Tables

**Table 1:** Morphological traits and scoring scheme used in the QBS-c framework for Collembola.

Trait	Trait Description	Class Description	Score	Eco-Morphological Interpretation
Body size	Maximum body length	>3.0 mm	0	Large, typically epigeic or weakly soil-adapted
		2.0–3.0 mm	2	Intermediate
		<2.0 mm	4	Small, associated with edaphic specialization
Pigmentation	Degree of body pigmentation	Complex pattern	0	Epigeic, exposed habitats
		Uniform coloration	1	Intermediate
		Weak or localized pigmentation	3	Reduced exposure to light
		Absent	6	Strongly euedaphic
Visual apparatus	Number of ocelli	8 + 8 ocelli	0	Fully developed visual system
		6 + 6 ocelli	2	Moderate reduction
		1 + 1 to 5 + 5 ocelli	3	Reduced visual capacity
		0 + 0 ocelli	6	Complete loss, euedaphic condition
Antennae length	Antennae/head diagonal ratio	>1.50	0	Long antennae, active surface mobility
		0.90–1.50	2	Intermediate
		0.50–0.89	3	Reduced
		<0.50	6	Strong reduction, soil-adapted
Leg length	Leg III/body length ratio	>0.60	0	Long legs, epigeic locomotion
		0.40–0.60	2	Intermediate
		0.20–0.39	3	Reduced locomotion
		<0.20 or reduced claw/empodium	6	Strongly reduced, euedaphic
Furca	Furca/body ratio and structure	>0.35	0	Well-developed jumping organ
		0.15–0.35	2	Intermediate
		0.05–0.14	3	Reduced
		Mucrone absent/modified dens-manubrium	5	Functionally reduced
		Absent or rudimentary	6	Fully euedaphic
Cuticular structures	External structures (setae, scales, etc.)	Rich macrochaetae/scales/trichobothria	0	Epigeic sensory structures
		Moderate coverage	1	Intermediate
		Localized specializations	3	Partial reduction
		Poorly developed or reduced	6	Euedaphic condition

## Data Availability

The original contributions presented in this study are included in the article/Supplementary Material. Further inquiries can be directed to the corresponding author.

## Supplementary Materials

The following supporting information can be downloaded at https://www.mdpi.com/article/10.3390/insects17070727/s1, Table S1: Summary of EMI attribution rules and special cases implemented in the QBS-ar interactive key; Table S2: Decision tree of the QBS-ar interactive identification key.
