# Supplementary material for: Soil Arthropod Diversity and Biological Soil Quality: A Web-Based Framework for Standardized EMI Attribution in QBS-ar and QBS-c
Source: Insects. 2026 Jul 14;17(7):727. doi: 10.3390/insects17070727 (PMC13409934; doi:10.3390/insects17070727)
Supplement: Supplementary file 1 [file insects-17-00727-s001.zip › insects-4426514-supplementary.pdf]

Table S1. Summary of EMI attribution rules and special cases implemented in the QBS-ar interactive key.

Taxonomic group	EMI assignment type	Rule description	EMI value(s)
Acari, Protura, Diplura, Palpigradi, Pseudoscorpiones, Pauropoda, Symphyla	Fixed	Strongly soil-adapted groups	20
Opiliones, Isopoda, larvae (Diptera, Hymenoptera, Lepidoptera, Mecoptera)	Fixed	Intermediate adaptation	10
Formicidae, Blattodea	Fixed	Moderate soil association	5
Adult Diptera, Hymenoptera, Psocoptera, Dermaptera	Fixed	Weak soil adaptation	1
Araneae	Variable	Size and pigmentation-dependent assignment	1–5
Collembola (general categories)	Variable	Gradient from epigeic to epi-edaphic forms	1, 2, 4, 6, 8
Collembola (clearly eu-edaphic)	Fixed (priority rule)	No pigmentation, no furca, reduced appendages	20
Coleoptera larvae	Fixed	Standard larval assignment	10
Coleoptera adults	Variable (cumulative)	Multiple traits (small size, wingless, thin cuticle, reduced eyes) contribute additively	1 or 5 + 5 + …
Orthoptera	Variable	Gryllidae vs other forms based on morphology	1 or 20
Hemiptera	Variable	Cicada larvae vs other hemipterans	10 or 1
Chilopoda	Variable	Size-based distinction (e.g., Geophilomorpha)	10 or 20
Diplopoda	Variable	Size-based distinction	10 or 20
Collembola (integration rule)	Special rule	Highest EMI retained across Collembola categories; euedaphic overrides others	max or 20
General QBS-ar calculation	Aggregation rule	Highest EMI per taxon retained, then summed across taxa	—

Table S2. Decision tree of the QBS-ar interactive identification key.

Node	Diagnostic character	Option	Output
N1	Body organization	Cephalothorax + abdomen	N2
		Segmented body	N6
N2	Constriction between body regions	Absent	N3
		Narrow waist	Araneae
N3	Abdomen segmentation	Present	N4
		Absent	Acarina
N4	Chelae	Present	Pseudoscorpiones
		Absent	N5
N5	Telson	Present	Palpigradi
		Absent	Opiliones
N6	Presence of legs	Absent	N7
		Three pairs	N10
		More than four pairs	N25
N7	Head capsule	Sclerotized	N8
		Not sclerotized	N9
N8	Mandibles	Articulated	Coleoptera larvae
		Hook-like/soft	Diptera larvae
N9	Head morphology	Sac-like	Hymenoptera larvae
		Internal/absent	Diptera larvae
N10	Prolegs	Present	N11
		Absent	N12
N11	Number of prolegs	≥6 pairs	Hymenoptera larvae
		≤5 pairs (crochets)	Lepidoptera larvae
		8 pairs	Mecoptera larvae
N12	Furca	Present	Collembola
		Absent	N13
N13	Mouthparts	Entognathous	N14
		Ectognathous	N15
N14	Antennae/cerci	Absent	Protura
		Present	Diplura
		Antennae only	Collembola (eu-edaphic)
N15	Halteres	Present	Diptera adults
		Absent	N16
N16	Elytra	Present	Coleoptera adults
		Absent	N17
N17	Abdomen type	Wingless pedunculate	Formicidae
		Winged pedunculate	Hymenoptera adults
		Not pedunculate	N18
N18	Cerci	Present	N19
		Absent	N20
N19	Cerci morphology	Forceps-like	Dermaptera
		Three appendages	Thysanura
		None	N20
N20	Hind legs	Jumping	Orthoptera
		Cursorial	Blattodea

Node	Diagnostic character	Option	Output
		No specialization	N21
N21	Foretarsi	Expanded	Embioptera
		Not expanded	N22
N22	Mouthparts type	Piercing-sucking	Hemiptera
		Chewing	N24
N24	Antennae	Long filiform	Psocoptera
		Short bead-like	Isopoda
		Variable	Coleoptera larvae
N25	Legs per segment	Two pairs	Diplopoda
		One pair	N26
N26	Forcipules	Present	Chilopoda
		Absent	N27
N27	Antennae/body size	Short, bifid	Pauropoda
		Long, filiform	Symphyla
		Flattened	Isopoda
